# Behaviour Support for People Living with Dementia in Residential Aged Care: A Cross-Sectional Survey of Staff Experiences and Support Needs

**DOI:** 10.1177/14713012251366740

**Published:** 2025-08-09

**Authors:** Alinka Fisher, Katrina Reschke, Nij Shah, Sau Chi Cheung, Claire M. C. O’Connor, Olivier Piguet

**Affiliations:** 1 Disability and Community Inclusion, College of Nursing and Health Sciences, 1065 Flinders University, Adelaide, SA, Australia; 2 School of Psychology and Brain & Mind Centre, 4334 The University of Sydney, Sydney, NSW, Australia; 3 Neuropsychology Unit, Royal Prince Alfred Hospital, Sydney, NSW, Australia; 4 School of Psychology, University of New South Wales, Sydney, NSW, Australia; 5 Neuroscience Research Australia, Sydney, NSW, Australia; 6 HammondCare, Centre for Positive Ageing, Sydney, NSW, Australia

**Keywords:** dementia, residential aged care, behaviour support, behavioural and psychological symptoms of dementia, support staff

## Abstract

**Objectives:** Behavioural and psychological symptoms of dementia (BPSD) are prevalent in residential aged care (RAC) settings, negatively impacting residents’ quality of life and increasing carer burden. This study investigated current practices and experiences of RAC staff in managing BPSD, including collaborative behaviour support planning with family members. **Methods:** A cross-sectional survey was conducted with 43 RAC staff supporting residents with dementia. Data was collected about demographics, resident characteristics, and current behaviour support practices and support needs. Quantitative and qualitative data were analysed using descriptive statistics and thematic analysis respectively. **Results:** Despite the prevalence of written behaviour support plans, their perceived usefulness and staff involvement in their development were inconsistent. Collaboration with family members is valued but often limited by factors such as time constraints and communication difficulties. Staff identified a need for increased training and additional resources to provide effective behaviour support. **Conclusion:** This study highlights the need for improved training and support for RAC staff in managing BPSD, and a practice framework that clarifies roles and responsibilities across behaviour support service systems. Further research is needed to inform evidence-based behavioural interventions in RAC settings, with emphasis on collaborative practices that support best outcomes for residents with dementia.

## Introduction

Over 400,000 people are living with dementia in Australia (AIHW, 2023), with approximately 188,000 in residential aged care (RAC) services (GEN Aged Care Data, 2023). Of those living in RAC services, 68% are reported to have high care needs arising from changes in cognition and behaviours (GEN Aged Care Data, 2023). This is not surprising given that behavioural and psychological symptoms of dementia (BPSD) are experienced by approximately 90% of people living with dementia throughout disease progression (Steinberg et al., 2006). These include verbal and physical aggression, property destruction, sexualised behaviours, apathy, wandering and self-harm (Cerejeira et al., 2012). The aetiology of BPSD is multifaceted and is the result of an interplay between biological (e.g., brain atrophy, cognitive deficits including communication impairments), psycho-social (e.g., personality) and environmental factors (e.g., living arrangements, unmet needs; Budson & Solomon, 2016).

BPSD may negatively affect a person’s relationships, community participation and overall quality of life. For the person with dementia, they are associated with a reduced quality of life (Ryu et al., 2011), decreased community participation, increased functional impairment (Cerejeira et al., 2012) and high emotional distress (Hiyoshi-Taniguchi et al., 2017). They also contribute to high levels of anxiety, depression, and burnout for family carers (Bakker et al., 2013; Hiyoshi-Taniguchi et al., 2017) and as a result, often contribute to an early transition to RAC services (Toot et al., 2016).

Non-pharmacological interventions (e.g., positive behaviour support [PBS]) are recommended as the first option in seeking to prevent and respond to BPSD (Dyer et al., 2018), specifically, proactive person-centred approaches that seek to understand and address the underlying reasons for the behaviour occurring. Here, the emphasis is on person-centred practices that support quality of life outcomes rather than solely reducing BPSD. Despite evidence demonstrating the effectiveness of behavioural approaches (Dyer et al., 2018) and the well-known limited efficacy and negative side-effects of pharmacological interventions (Piguet et al., 2017), pharmacological interventions are still widely used to manage behaviours (known as ‘chemical restraint’). The Royal Commission into Aged Care has revealed an overreliance on chemical restraints in RAC services, followed by urgent recommendations for systems reform and person-centred behaviour support practices that respect a person’s rights and dignity (Royal Commission into Aged Care Quality and Safety, 2021).

Australian legislation now requires clinicians using regulated restrictive practices (including chemical and physical restraint) to develop an individualised behaviour support plan for the individual in their care (Aged Care Act 1997, 2023). However, no specific guidelines exist around who (i.e., what profession) and what skills or practice models are required to inform the development of these plans. By comparison, under the National Disability Insurance Scheme (NDIS), individualised behaviour support plans are to be developed by NDIS behaviour support practitioners, where there is a specific role and defined capabilities required to conduct this specialist work (NDIS Quality and Safeguards Commission, 2019). Indeed, individualised behaviour support requires technical knowledge and skills in analysing situations and problem-solving solutions, but also soft skills required to work with the person and other key stakeholders to inform a collaborative and person-centred process (e.g., see Fisher, Louise, et al., 2024). As reported by Kelly et al. (2024), this often involves negotiating shared, and sometimes conflicting goals between the person, family members, and support providers.

In a RAC setting, support staff are responsible for providing daily care and support for residents and are likely to be key players in managing BPSD. Despite the high occurrence of people living with dementia with significant behaviour support needs in RAC settings, and the key role of RAC staff in being responsive to these support needs, research suggests that staff receive limited training specific to behaviour support. For example, in a recent pilot study by Fisher, Reschke, et al. (2024), many RAC staff who attended introductory PBS training reported this to be the first time they had received education specific to behaviour support. The training covered topics such as identifying and addressing the underlying reasons for BPSD and was delivered through a series of behaviour support workshops that included both support staff and family members to facilitate a collaborative approach to behaviour support planning and implementation (Fisher, Reschke, et al., 2024). This approach was underpinned by evidence that family involvement in behavioural intervention for people living with dementia has been associated with improved outcomes for the person and support staff (de Oliveira et al., 2019; O'Connor et al., 2021). Despite the benefits of collaborative practice and the negative impacts related to BPSD, there appears to be limited literature examining collaborative behaviour support practices and how this can be effectively facilitated in RAC settings.

This study examined current practices and experiences of RAC staff providing behaviour support to people living with dementia in RAC services, including exploring preliminary insights into collaborative behaviour support practices with family members. These insights are crucial as we consider strengths and skill gaps within the current workforce, with implications for policy and practice improvements as we seek to inform more efficient and effective behaviour support services that prioritise the quality of life of people living with dementia.

## Method

### Survey Design

A cross-sectional survey was designed to gather insights into current practices and experiences of those providing behaviour support to residents with dementia in RAC services. This included 10 questions relating to demographic characteristics of staff members (e.g., age, sex, years of experience, qualifications, and training) and 11 questions about residents they support with dementia. This included their dementia diagnosis and type, the frequency, and the impact of BPSD (using a detailed list informed by the Overt Behaviour Scale; Kelly et al., 2006), and whether they had behaviour support plans in place). Of the remaining questions, 11 were specific to current practices, including behaviour support strategies being used, who is responsible for developing individualised behaviour support plans, and the facilitators and barriers to providing effective support. Finally, four questions elicited information about the benefits and challenges of collaborating with family members to inform behaviour support practices.

### Data Collection

This research project was conducted in accordance with the Helsinki Declaration and was approved by the Flinders University Human Research Ethics Committee (project number: 5373). The survey was administered online using Qualtrics software (Qualtrics Labs Inc., Provo, USA). Inclusion criteria required that respondents be over the age of 18 and were in a paid role (e.g., support worker, nurse) providing direct support to residents with dementia in RAC services in Australia. The direct link to the survey was distributed via social media platforms, including LinkedIn and Twitter, and ‘tagged’ (‘@’) professional bodies associated with dementia and nursing agencies such as the Aged and Community Care Providers Association (ACCPA), Australian Nursing and Midwifery Journal and Dementia Australia. Additionally, RAC services (identified using the Australian Government’s My Aged Care website) were contacted via email with study details and asked to circulate information about this study (information sheet and flyer with direct link to survey) to paid staff who support people with dementia within their RAC service. A snowball sampling approach was also used, with respondents being asked to forward the survey to contacts who also met inclusion criteria.

Due to slow uptake of the survey, following an ethics modification request, the closing date was extended by two weeks, and a flexible convenience-sampling approach was adopted. This involved identifying RAC services (via the My Aged Care website) in inner metropolitan areas in Adelaide (South Australia) and Sydney (New South Wales) where the research team was located, and personally delivering hard copies of the survey flyer (with a QR code to the survey). This included a total of 38 RAC services (28 in Adelaide, 10 in Sydney), where reception staff were requested to place study flyers within the staff room (or where most easily accessible to staff). An additional 27 RAC services were contacted via email and asked to print and/or circulate the flyer to support staff.

### Data Analysis

Quantitative results were analysed using descriptive statistics. This included 31 closed ended questions (e.g., multiple choice, Likert-type, and sliding-scales [e.g., 0–100%] to quantify responses). The questions with a sliding scale were used to quantify their responses or approximate their responses as ‘percentages’ (e.g., when asked to indicate the % of clients with BPSD or written behaviour support plans). The sliding scale, despite using increments of ‘5' or ‘10' recorded these responses as the scaled count (e.g., ‘42' instead of ‘40' or ‘45'). This resultant inaccuracy of the Qualtrics survey sliding scale tool influenced the way data has been reported in this paper.

Reflective Thematic Analysis (RTA; Braun & Clarke, 2022) was used for the responses to five open-ended questions to extract and summarise key experiences and support needs. This method allowed for flexibility in interpreting nuanced qualitative data while acknowledging the researchers’ role in meaning-making. Thematic analysis was conducted independently by both the second and third authors (NS and KR) through line-by-line coding to label segments, which were then grouped into categories and synthesised into broad conceptual categories relevant to research objectives. Throughout this process, reflective discussions were held among the research team (authors AF, NS and KR) to ensure the analysis was representative of the data and to acknowledge potential influences on interpretation resulting from respective backgrounds as clinicians and researchers with experience in PBS and dementia care.

Thematic analysis was used for responses to five open-ended questions (Braun & Clarke, 2022) to extract and summarise key experiences and support needs. Thematic analysis was conducted independently by both the second and third authors (NS and KR), including line-by-line coding to label segments, which were then grouped into categories and synthesised into broader conceptual categories relevant to research objectives. This process also included reflective discussion between the research team (authors AF, NS and KR) to ensure analysis was representative of the data.

## Results

### Respondent Characteristics

A total of 66 online survey responses were recorded over 2 months by Qualtrics analytics with 43 datasets included for analysis (including respondents who completed all demographic questions and submitted the survey [partial or full completion]). Demographic details were recorded for 43 RAC staff and are presented in Table 1. A majority (74%; n = 32) identified as female and 60% (n = 26) worked in New South Wales. Respondent ages ranged from 18 to 66+ years; 44% were 26-45 years and 44% were 46-66+ years. The highest level of completed education of respondents was a Certificate/Diploma /Advanced Diploma for 47 % (n = 20), followed by a Bachelor degree/Honours (23%; n = 10). With regard to their current role, 44% (n = 19) described their role as Support/Aged Care Workers, 28% (n = 12) were Allied Health Professionals, 9% (n = 4) were Facility/House Managers, and 9% (n = 4) were Lifestyle Coordinators. Of the remaining, 7% (n = 3) were nurses and one (2%) was a Team Leader. Respondent nominated qualifications were Assistants in Nursing (16%; n = 7), Registered Nurses (9%; n = 4), or support workers (Certificate III/IV in disability) (23%, n = 10). Those who responded ‘Other’ (23%; n = 10) included ‘Diploma of Leisure and Health’, ‘Advanced Diploma in Volunteer Management’ and ‘Cert IV in Screen and Media’, with one describing their role as a ‘Behaviour Analyst’. For most respondents (84%; n = 36), English was their primary language with the remainder having Cantonese as their primary language (16%; n = 7).

**Table 1.** Respondent characteristics

Variable and level	
Gender, n (%)	
Male	7 (16%)
Female	32 (74%)
Non-binary/third gender	0 (0%)
Prefer not to say	1 (2%)
Age (years), n (%)	
18-25	5 (12%)
26-35	10 (23%)
36-45	9 (21%)
46-55	6 (14%)
56-65	12 (28%)
66+	2 (1%)
State/Territory of work, n (%)	
New South Wales	26 (60%)
Victoria	7 (3%)
South Australia	10 (23%)
Queensland	2 (5%)
Western Australia	5 (2%)
Highest level of education completed n (%)	
Secondary school not completed	4 9%)
Secondary school completed	4 (9%)
Certificate / Diploma / Advanced diploma	20 (47%)
Bachelor’s degree / Honours	10 (23%)
Postgraduate degree	5 (12%)
Qualification, n (%)	
Support worker (Cert III/IV in disability)	10 (23%)
Assistant in nursing	7 (16%)
Registered nurse	4 (9%)
Psychologist	5 (12%)
Occupation therapist	1 (2%)
Exercise Physiologist	1 (2%)
Developmental Educator	3 (7%)
Social worker	1 (2%)
Other	10 (23%)
Current role, n (%)	
Support worker/Aged care worker	19 (44%)
Allied health professional	12 (28%)
Facility/House manager	4 (9%)
Lifestyle Coordinator	4 (9%)
Nurse	3 (7%)
Team Leader	1 (2%)

Respondents’ years of experience providing support to people with dementia ranged from 1 to 42 years with 60% (n = 22) 5 years or less experience (mode of 3 years, median of 5 years). In the previous 3 years (prior to completing the survey), a majority of respondents (69%; n = 22) had received no more than one day of training specific to behaviour support for people with dementia (excluding medication and pharmacological treatment), with 38% (n = 12) having received 1-4 hours, and 31% (n = 10) receiving 5-8 hours of training. Of the remaining respondents, 16% (n = 5) received 1-2 days of training in behaviour support, 9% (n = 3) had 17+ hours of training, and 6% (n = 2) had not participated in any training during this period.

### Residents with Dementia and BPSD

Respondents reported to be supporting residents with various dementia types, with different subtypes indicated by varying proportions of respondents. Alzheimer’s disease was reported the most frequently (76%), followed by dementia aetiology not specified (62%), mild cognitive impairment (54%), mixed type (e.g., vascular dementia and Alzheimer’s disease together; 43%), vascular dementia (41%), frontotemporal dementia (32%), Lewy body dementia (22%), and ‘query dementia’ (but no formal diagnosis; 14%). One respondent (3%) selected ‘other’ indicating ‘dementia as well as psychological disorders’. When asked how many residents with dementia they supported during a typical two-week period, responses ranged from 2 to 50 people, with a mean of 18 per fortnight.

When asked what percentage of residents with dementia they supported presented with BPSD, the most common response (mode) was 100% (reported by 19% of participants), with responses varying from 2% to 100% (average 44.5%). They then indicated what specific BPSD they observe and manage in the people they support with dementia. Several types of BPSD were reported, with the most frequent being verbal aggression (96%), followed by repetitive behaviours (92%), wandering (88%), physical aggression against others (75%), inappropriate social behaviour (71%) and reduced initiation/apathy (67%). Inappropriate sexual behaviour (42%) and physical acts against self (29%) were less commonly reported. Respondents were then asked to indicate which of these behaviours they found to be most difficult to manage. Of the 25 (58 %) who responded to this question, physical aggression was reported as the most difficult to manage by 28% (n = 7), followed by verbal aggression (20%; n = 5), inappropriate social behaviour (20%; n = 5), perseveration/repetitive behaviour (20%; n = 5), and wandering and absconding (16%; n = 4). Respondents reported these behaviours to occur from at least once daily (40%), to once a week or more (16%) about a month (28%) or less often (16%). Nearly half of the 25 respondents reported this behaviour caused them moderate levels of stress/worry (48%; n = 12).

### Current Behaviour Support Practices

Respondents were asked to indicate the ‘most common’ behaviour support approach for people with dementia and BPSD in their RAC service. Of the 29 who responded to this question, 66% (n = 19) reported that residents with dementia and BPSD typically had written behaviour support strategies included in the person’s Care Plan (i.e., an integrated support plan not specific to BPSD) and 28% (n = 8) indicated that a more targeted behaviour support process (e.g., with support from Dementia Support Australia; a nationwide outreach behaviour support service) was common, including the development of a written behaviour support plan. They were then asked to estimate the percentage of the people they supported with dementia who received medication to manage behaviours (i.e., to treat BPSD), and of the 32 RAC staff who responded 22% (n = 7) indicated this to be true for approximately 41–50%. Remaining responses varied, with estimates of 20–100% of people with dementia in RAC services receiving medication for this purpose.

Regarding their ‘usual’ approach to managing BPSD, 59% of respondents (n = 19) reported using the recommended strategies in the person’s behaviour support plan (i.e., from a formal/written support plan) and 31% (n = 10) reported applying strategies they knew to be helpful to de-escalate but that had not been recommended/provided in a formal written support plan. None of the respondents reported medication as their usual (‘go to’) approach to reduce agitation and risk of harm. When these respondents were asked what they usually do to reduce the chances of BPSD occurring, a majority (78%; n = 25) indicated a focus on using preventative strategies from a resident’s formal/written behaviour support plan (e.g., tailored care and environmental improvements informed by their preferences). Five (16%) respondents reported they usually used strategies they had learnt were helpful but had not been recommended/provided in a plan and 2 respondents indicated they usually provided medication to prevent the occurrence of BPSD.

Respondents were also asked to indicate their level of confidence in both identifying and implementing effective behaviour support strategies according to two 5-item multiple choice questions. Nine respondents (41%) reported being confident in identifying effective strategies (responding ‘yes’ to this question), whilst seven respondents (32%) reported being ‘somewhat’ confident and 27% (n = 6) ‘moderately’ confident in this domain. When respondents were asked whether they felt confident in implementing behaviour support strategies, 50% (n = 11) who responded to this question indicated ‘yes’, whilst 18% (n = 4) reported ‘moderately’ and 32% (n = 7) reported to be ‘somewhat’ confident in implementing behaviour support strategies.

### Written Behaviour Support Plans

When asked whether the behaviour support plans developed for residents with dementia were helpful in providing effective support, a majority (52%; n = 15) indicated ‘sometimes’ and almost one third (28%; n = 8) indicated ‘rarely’. Conversely, only 6 respondents (21%) indicated them to be ‘always’ helpful. Respondents were also asked whether behaviour support plans included individualised strategies focused on improving the person’s environment and/or reactive strategies. ‘Environmental’ strategies were defined as those focused on modifying the physical and/or social environments around a person (e.g., structural changes, communication strategies). ‘Reactive’ strategies were defined as those that outlined how to respond/react to the occurrence of the BPSD, for example incident/crisis management strategies that may include administration of medication. A majority of respondents (55%; n = 16) indicated behaviour support plans ‘sometimes’ include individualised strategies to improve the person’s physical and/or social environment and similarly ‘sometimes’ included reactive strategies (59%; n = 17).

**Figure 1.**
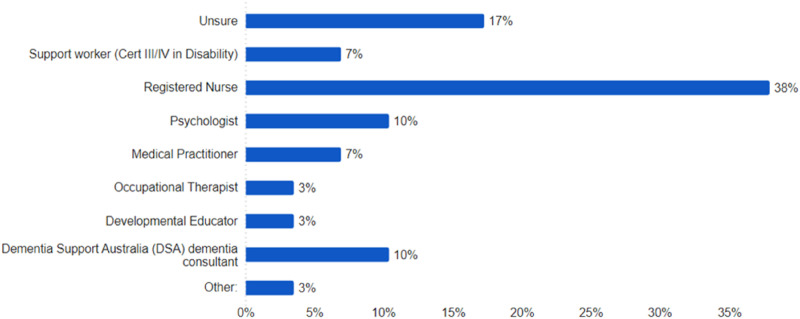
Participant Perceptions Around what Roles are Involved in D*evelopment of written behaviour support plans*

According to over a third of respondents (38%; n = 11), registered nurses were mostly responsible for developing/writing behaviour support plans, with 17% (n = 5) ‘unsure’ who carried out this role. Of the remaining, varied professionals were reported to develop these plans, including allied health professionals, Dementia Support Australia (DSA) consultants and support workers (Figure 1).

There was great variability in how involved respondents (RAC staff) were in the development of written behaviour support plans (e.g., being consulted, informing development of strategies). The majority of respondents (56%; n = 16) reported they were ‘always’ or ‘sometimes’ involved, compared to 44% (n = 13) who reported being ‘never’ or ‘rarely’ involved in this process.

### Collaborating with Family Members

Respondents were asked to indicate the level of involvement of family members in behaviour support planning for residents with dementia in their RAC service. Of 22 respondents, 95% reported family members had some level of involvement; with half (n = 10) rating moderate involvement (e.g., discuss strategies); 27% (n = 6) have mild involvement (e.g., some consultation); and 18% (n = 4) having major involvement (e.g., discuss strategies, problem-solve solutions together and modifying plans as needed).

Respondents were then asked to indicate any benefits to collaborating with family members around behaviour support planning for residents with dementia. Of the 18 responses to this question, one overarching theme was identified around improved outcomes.

#### Family Collaboration Supports Improved Outcomes

Staff emphasised the benefits of utilising the families’ understanding and knowledge of the person with dementia, with this supporting a collaborative approach in developing appropriate support strategies. For example, one staff member commented that families “*often have detail on how the person’s behaviour has changed and what strengths / memories / positive attributes the person brings that can be used to reduce behaviours and encourage quality of life.” (P28*). Others commented that by working together *“the care staff and family can come up with a way they are both comfortable to reduce this behaviour” (P9)* and that family collaboration is *“Definitely beneficial in providing a holistic perspective” (P28)*.

Respondents were also asked about any barriers to family collaboration.

#### Challenges Around Family Working with Families

Responses emphasised challenges relating to family members having limited education or understanding around dementia and behaviour changes, in addition to limited time and/or disinterest in being involved. For example, one respondent commented that *“Some families don’t recognise that their family member has behaviours of concern” (P10*) and another suggested that *“Some family members not having time or just not wanting to be there” (P5)*.

Other comments also highlighted differing perspectives, attitudes, and approaches to behaviour support. For example, one respondent suggested that family members *“are in denial about the condition of their loved one” (P8)* and another indicated that family members *“…may not agree to something even though the care staff know it will best support them” (P9)*. There were concerns raised that family members felt that “*the behaviours are only the result of the surrounding environment and not self-perpetuating” (P7)* and are *“worried strategies won’t work due to the changing and degenerative nature of the disease” (P28)*.

### Barriers to Providing Effective Behaviour Support

In response to an open-ended question eliciting information about their current challenges and barriers when providing behaviour support to people with dementia, two key themes were identified: ‘lack of knowledge and skills’ and ‘insufficient time and resources’.

#### Lack of Knowledge and Skills

Of the 20 who responded to this question, 12 reported barriers related to inexperienced and unskilled staff. For example, respondents reported: *“Staff with insufficient knowledge of the resident in question” (P7)*, and *“inexperience (newly qualified Aged Care Support Worker)” (P12)*. Respondents also specifically reported that a lack of knowledge and understanding about behaviour and behaviour support processes was a barrier, *“Staff not agreeing with level of support, not understanding the situation or saying ‘that’s just them’ and therefore accepting the behaviour as normal” (P36)*, and the *“strategy plan has no effect” (P16)*. One respondent also reported the barrier of *“not being involved in clinical care planning” (P10)*.

#### Insufficient Time and Resources

Seven respondents reported barriers relating to time and resourcing, including insufficient time needed to provide effective behaviour support and insufficient staff needed to meet support needs. For example, one respondent emphasised *“Lack of staff around dementia area is not working well. It’s a big challenge when you look after 12 - 16 people with dementia with one or two staff in one area to provide good behaviour support” (P6)*. Another commented on the barrier of *“*[lack of] *time, support from management, support from GP, lack of staff” (P13)*. Other respondents acknowledged that support for a person with dementia demands additional allocated time with *“the biggest barrier … often time and the changing nature of dementia.” (P36)* and *“Not enough time to provide support required for each person” (P8)*.

### Support Needs and Preferences

When asked to comment on what supports would help them in providing effective behaviour support, key themes directly reflected barriers identified above. These included the need fo*r ‘training that incorporates general positive behaviour strategies for people with dementia’ (P36)* and *‘enough time … more staff’ (P15)*.

#### Training and Education

Of 23 responses, 8 emphasised the usefulness of further training and collaborative education in behaviour support. One respondent for example, surmised that “*From my experience, aged care workers are not often as familiar with positive behaviour practices as the disability industry.” “...I really think many workers would benefit from training that incorporates general positive behaviour strategies for people with dementia on an ongoing basis to ensure best practices and continuity of care” (P28)*. Other respondents commented that *“Information, updates regarding behavioural management strategies” (P4)* and “*Support from other staff members on how to approach the individual situations from their own experiences as a care support worker” (P9)*, would be supportive.

More resources: Eight respondents indicated that increased staff numbers and more time would help them provide effective behaviour support. For example, respondents suggested *“Better staff to resident ratios” (P8)* and *“more staff more activities more time for 1:1 time” (P2)*.

Two staff also indicated the need for “*having the family more involved” (P24)* and that *“family support” (P5)* would help when providing behaviour support.

### Training Materials and Delivery Preferences

Respondents were asked to think about their preferences for training program delivery. A range of training options were selected by respondents with ‘behaviour support education and resources for family members’ the most preferred training option selected by 59% (n = 13) of respondents. ‘In-person training’ (e.g., group workshop) and ‘a combination of in-person training (e.g., group workshop) and online materials (modules)’ were each preferred by 50% (n = 11) of respondents, ‘online short courses’ e.g., 1-3 hrs 45% (n = 10); ‘on-site training/coaching’ 41% (n = 9); ‘video examples’ 36% (n = 8); and ‘reading material’ 31% (n = 7) were also included as helpful training modes. One respondent recommended ‘other’ training modes (14%, n = 3) suggesting that *“Behaviour support education and resources for manager” (P16)* would also be helpful in providing effective behaviour support.

## Discussion

The outcomes from this survey provide valuable insights into current behaviour support practices for people with dementia in RAC services in Australia. The findings emphasise the high prevalence and challenges associated with BPSD for this population and the critical need for effective support strategies. Our results, however, also highlight inconsistencies and challenges in the delivery of such services.

It was promising that staff reported many residents with dementia to have documented behaviour support strategies in their Care Plan, with some also having specific behaviour support plans. Unfortunately, however, a significant proportion of respondents reported these plans to be only ‘sometimes’ or ‘rarely’ helpful. Moreover, nearly half of respondents indicated that they were not involved in the development of these plans. This is concerning, as reported by Fisher et al. (2022), the involvement of those providing direct support, given their primary role implementing behaviour support strategies, is crucial for ensuring behaviour support plans are relevant and effective. A lack of involvement, not only risks plan quality, but may lead to a generalised “plan apathy” (disengagement) among staff and potentially compromise the fidelity of implementation (Fisher et al., 2022).

The survey also revealed a lack of clarity regarding roles and responsibilities for developing behaviour support plans. Over one third indicated that registered nurses were responsible, whilst others were ‘unsure’ or reported plans to be developed by varied allied health professionals, Dementia Support Australia consultants (who provide outreach behaviour management services) and even support workers. This inconsistency reflects the need for more standardised guidelines and clear delineations of roles within the RAC service sector. Indeed, current legislation now requires the development of behaviour support plans (Aged Care Act 1997, 2023), but there is no guidance about who is responsible or is expected to have the skills to carry out this specialist work and using what practice model. In an attempt to understand the behaviour support practitioner workforce in Australia, Kelly et al. (2024) conducted a national survey of those responsible for developing behaviour support plans, including for those with dementia in RAC services. However, despite targeted recruitment efforts across the aged care sector, of the 492 respondents, only 0.9% (n = 4) reported to be developing behaviour support plans for people with dementia. This does not only raise the question of who is doing this work, but also who identifies as being *responsible for* this work. Furthermore, in a recent study conducted by Wesson et al. (2024), 64 behaviour support plans were analysed across seven providers (24 sites) across eastern Australian states, which revealed variability in the specificity of behaviour support plans. This again emphasises inconsistency regarding behaviour support practices and the urgent need for further behaviour support guidance and education for the sector.

Interestingly, some staff reported that behaviour support plans were developed by specialist and resource-intensive outreach services such as Dementia Support Australia’s Dementia Behaviour Management Advisory Service (DBMAS). This is not surprising given reports of DBMAS supporting 68,500 referrals between 2017 – 2023, and with 87% of these originating from RAC services. However, it is concerning that 87.8% relate to mild-to-moderate BPSD (Macfarlane et al., 2021), which speaks to the need to develop the necessary expertise and practice leadership within RAC services to build capable and responsive support systems. Indeed, the outsourcing of this specialisation not only places a burden on the health care system, but also suggests a crisis-driven and reactive approach that is concerning. Simply, the current service system reacts to the occurrence of BPSD after they manifest, rather than seeking to prevent them from happening. It is essential to shift towards a proactive model that prioritises person-centred care and addresses underlying environmental factors that may contribute to BPSD.

The inconsistent elements of behaviour support plans being developed (e.g., that ‘sometimes’ include environmental and or reactive strategies), and the confusion surrounding who is responsible for developing these plans emphasises the need for a clarified behaviour support service model. This would ensure a consistent understanding of the required practices, competencies, and roles within the system. For example, a tiered approach, as proposed by Fisher and Kelly (2024) could help to establish minimum expectations (e.g., the provisions of adequate environments and person-centred approaches to care) and reserve more specialised supports for when it is truly needed. This may include targeted assessment, formulation, planning and implementation support (Fisher, Louise, et al., 2024) for when the relationship between BPSD, the person and their environment is unclear (Fisher & Kelly, 2024).

A key barrier to providing effective behaviour support to people with dementia in RAC services identified in this study was the lack of training and expertise among staff. Many respondents reported receiving limited training specific to behaviour support for people with dementia and expressed a lack of knowledge and skills. This reflects similar findings from a recent PBS training pilot in RAC services (Fisher, Reschke, et al., 2024), with some staff reporting that this pilot (which included a 1-day introductory workshop for support staff) was the first time they had received specific training in behaviour support planning.

An inadequate understanding of individualised behaviour support may lead to unrealistic expectations and challenges in implementation. For example, this may explain why it is expected that registered nurses lead and oversee behaviour support planning without having the necessary specialised training. Further, the primary medicalised training of nurses, who are often responsible for developing behaviour support plans may in part explain concerns regarding the over-reliance on pharmacological approaches (Westbury et al., 2018). The Aged Care Royal Commission’s recommendations for systems reform and increased training in person-centred and non-pharmacological approaches (Royal Commission into Aged Care Quality and Safety, 2021) are essential to address these issues.

In addition to limited training, staff reported insufficient time and resources as barriers to providing effective behaviour support. This again aligns with findings from the PBS training pilot (Fisher, Reschke, et al., 2024), which suggested that competing priorities and time constraints can contribute to disengagement among staff. Further, research suggests that staff often feel rushed to deliver care in RAC settings (O'Connell et al., 2021) and that the implementation of new evidence-based practice (e.g., the development of individualised behaviour support plans), may conflict and compete with existing and constantly shifting practice priorities (Masso et al., 2014). These factors raise important questions about the feasibility of expecting staff within RAC services to develop specialist behaviour support plans alongside their other responsibilities. It also emphasises the need for systemic changes to support the implementation of new policies that require practice change.

This survey was the first known to the authors that specifically sought to gain insights regarding the practices and attitudes of staff towards collaborative behaviour support, including collaboration with family members. This is noteworthy, especially given the recommendation for families to be involved in behaviour support planning in the Clinical Practice Guidelines and Principles of Care for People with Dementia (Guideline Adaptation Committee, 2016). It was promising that a large majority of staff indicated that family members were involved in behaviour support planning and reported benefits of a collaborative approach, with family involvement leading to more effective strategies. However, staff also reported barriers related to the families’ limited understanding around BPSD, poor communication, lack of time and even ‘disinterest’ in being involved. It is possible, however, that this is in part due to systems not being in place that enabled or welcomed this involvement. For example, in the recent PBS training pilot conducted by Fisher, Reschke, et al. (2024) some families felt their contributions were not valued, and recommendations were made for establishing a culture and systems that supported these collaborative behaviour support practices.

### Recommendations

The findings of this survey inform several practice and research recommendations in establishing consistent and high-quality behaviour support for people with dementia living in RAC services. These include the need for:

• Further dementia-specific behaviour support training for staff in RAC services, grounded in theoretical frameworks and person-centred practices.
• Examining the benefits and barriers to establishing a dedicated role for behaviour support practitioners (those responsible for developing and guiding implementation of behaviour support plans), who have specialised expertise in behavioural assessment, formulation, and planning.
• An evidence-based practice framework that outlines roles, responsibilities, and expectations within the behaviour support service system.
• Research to evaluate the current landscape of behaviour support services to inform policy and service development.
• Establishing collaborative practices that foster effective communication and enable practices that leverage collective expertise and experiences to support best outcomes for the resident with dementia. This includes collaborating with family and multidisciplinary teams to support the development of behaviour support plans and their implementation.

### Strengths and Limitations

A strength of this study was its design, which allowed us to both quantify experiences and gain insights through open-ended questions. This is also one of the first studies to examine the experiences of RAC staff providing behavioural support to people with dementia in Australian RAC services, and with a specific focus on examining benefits and challenges of collaborative behaviour support practices with family members.

Some limitations, however, must be acknowledged. Arguably, the sample may not be representative of the broad population of care support staff working with people diagnosed with dementia. As such, participation bias is unknown. For example, respondents with negative experiences or a strong commitment to behaviour support may be more likely to participate. Also, despite clear communication of the confidential and anonymous nature of the survey, there is a risk that participants’ responses may have been influenced by practice obligations, such as the requirements to use a least restrictive approach, rather than reflecting actual practice.

Furthermore, while respondent demographics varied across roles and backgrounds, reflecting the diverse and often inconsistent nature of this field, respondents were primarily English-speaking. This is not fully representative of the cultural and linguistic diversity within RAC services. Indeed, in 2021, the Australian Institute of Health and Welfare reported that over 51% of residential aged care workers were born overseas, with two in five (39%) speaking a language other than English at home (AIHW, 2025). The lack of linguistic diversity within this survey possibly stems from the small response rate and the survey being administered in written English. This limitation may make it difficult to generalise our survey findings to all of those who routinely provide behaviour support to residents with dementia in RAC. This is an area that warrants targeted investigation, as person-centred supports rely on effective communication with both the individual and their support team.

## Conclusions

Aged Care is a rapidly growing sector in Australia, and given the increasing prevalence of dementia and BPSD, the establishment of effective and sustainable behaviour support systems is a national priority. This study provides important insights into the current state of behaviour support practice in RAC services and highlights the need for significant improvements.

The recommendations we propose emphasise the importance of establishing a clear behaviour support practice model with defined roles, responsibilities, and communication pathways. This will require policy and funding changes to support the development of workforce capabilities and quality improvement initiatives. Ultimately, these recommendations seek to enhance the well-being of residents with dementia and staff in RAC services and contribute to a more efficient and effective service system that promotes consistent and high-quality care.
